# Emotional (dys)Regulation and Family Environment in (non)Clinical Adolescents’ Internalizing Problems: The Mediating Role of Well-Being

**DOI:** 10.3389/fpsyg.2022.703762

**Published:** 2022-03-31

**Authors:** Beatriz Raposo, Rita Francisco

**Affiliations:** Católica Research Centre for Psychological - Family and Social Wellbeing (CRC-W), Universidade Católica Portuguesa, Lisboa, Portugal

**Keywords:** emotional regulation difficulties, family environment, adolescence, well-being, internalizing problems

## Abstract

Adolescence is a period of several changes and a time when young people are confronted with some difficult tasks of dealing with a diversity of emotions and building their own identity. Therefore, it is a period of higher vulnerability for the development of internalizing problems. The present paper aims to study some constructs considered relevant to adolescents’ adjustment and/or internalizing disorders, emphasizing the role of well-being, emotional regulation and family environment. Therefore, this research aims to (1) test the mediating role of well-being in the relationship between emotional regulation difficulties, the family environment, and internalizing problems, and (2) understand the differences between adolescents with a higher and lower risk of presenting internalizing problems. In the study, 723 adolescents of both sexes (12–18 years old) from middle to high school completed self-report questionnaires. The results indicated that the mediating role of well-being was partially established between emotional regulation difficulties and internalizing problems, explaining 31% of the variance in these problems. Well-being was also considered a partial mediator between family environment (cohesion and support and conflict) and internalizing problems, explaining 19 and 26% of the variance, respectively. Furthermore, the group with a higher risk of developing internalizing problems (*n* = 130) revealed higher levels of emotional regulation difficulties and family conflict. In contrast, this group reported less family cohesion and support and lower levels of well-being. The main results of the present study provide relevant data in the context of clinical practice. Important implications are also discussed for the design of psychopathology prevention programs and the promotion of global well-being with adolescents. Considering the limitations of the present study, such as the nonrandom sampling process and the reduced number of participants included in the clinical group, these results need to be deepened in future research in this area.

## Introduction

Adolescence is a very important period in the individual’s development process and the consolidation of his or her autonomy, involving biological, psychological, cultural and psychosocial changes (Parsons, 2003). In this sense, adolescence is a vulnerable period for developing mental health problems (Costello et al., 2003). The World Health Organization (WHO, 2019) alerts that 20% of adolescents suffer from some mental disorder, and approximately half of them initiate it at approximately 14 years old. Studies carried out in several countries reveal that depression, anxiety, and eating disorders are the most prevalent mental disorders in the young population (e.g., Caldas de Almeida and Xavier, 2009; Roberts et al., 2009). These disorders are often grouped into “internalizing disorders,” as they are based on excessive impulse control and are manifested through various symptoms of anxiety, depression, social isolation and somatic complaints. Usually, they express toward the individual and not toward others, contrary to “externalizing disorders” (Cosgrove et al., 2011).

Some constructs are considered relevant in adolescents’ psychosocial adjustment, emphasizing the role that well-being can play in protecting against the development of internalizing disorders. Well-being is an innovative construct in this area, which still needs much research concerning other factors that are still more studied. Therefore, the present study intends to understand the role of well-being in the relationship of two important factors previously identified as relevant to the development of internalizing disorders (emotional regulation difficulties and family environment). This will allow, in the future, an understanding of how the promotion of well-being can contribute to the prevention of internalizing disorders and the improvement of adolescents’ psychosocial adjustment.

Subjective well-being encompasses three dimensions—social, psychological, and emotional—considered independent but interrelating and impacting mental health (Keyes and Waterman, 2003). Social well-being refers to one’s self-assessment of the quality of relationships with others and with the community in which they are inserted, focusing on the social functioning of the subjects from the point of view of commitment and social integration. Psychological well-being refers to positive self-assessment and self-acceptance and one’s continuous personal development process, setting goals and perceiving the meaning and purpose of life (Keyes, 2002). Emotional well-being encompasses the individual’s emotions and satisfaction evaluation concerning general and specific areas of their own life (Keyes and Waterman, 2003). Considering the difficulties associated with adolescence, it is important to explore and identify what contributes to adolescents’ subjective well-being of. Based on the tripartite model of the subjective well-being by Keyes (2002), a happy teenager is allegedly cognitively satisfied with life and experiences positive emotions (excitement, happiness) more frequently than negative emotions (nervousness, anger and anguish) (Martin and Huebner, 2007).

The identification of protective and risk factors associated with adolescents’ subjective well-being is extremely important to understand what influences the lives of young people (Cunsolo, 2017). Several studies show that low levels of psychological well-being, namely, with adolescents (Melo and Mota, 2014), are associated with symptoms of anxiety and depression (Derdikman-Eiron et al., 2011; Khazanov and Ruscio, 2016), with individuals with low levels of psychological well-being being even more likely to develop depression in the following 10 years (Wood and Joseph, 2010). The decline in general well-being at the beginning of adolescence may be due to feelings of stress that fall under the phase transition during this life stage, with a special focus on difficulties in the relationship between peers and in love relationships, the transition of basic education to secondary education, increased academic demands, and social pressure (Abela and Hankin, 2008). In contrast, the end of adolescence corresponds to a period of growing maturity and progression of the bylaws and a reduction of the anguish felt at the beginning of adolescence (Ge et al., 2006). In this sense, involving adolescents in positive psychology interventions has great potential as it can significantly enhance well-being and decrease symptoms of internalizing problems in both clinical and nonclinical groups (e.g., Sin and Lyubomirsky, 2009; Ng and Ong, 2022).

The theoretical and empirical conceptualizations indicate that emotional regulation difficulties play a critical role in developing and maintaining depression and anxiety symptoms in adolescence (McLaughlin et al., 2011; Ahmed et al., 2015; Schäfer et al., 2016; Beveren et al., 2019). Emotional regulation refers to various conscious and unconscious processes that can be implemented at different stages of the emotion generating process, affecting emotions’ occurrence, intensity, duration, and expression (Gross, 2002; Thompson et al., 2008). Thus, it refers to a set of strategies that the individual uses to increase, maintain or decrease one or more components of a certain emotional response, namely, at the physiological, cognitive, behavioral, experiential and social levels (Gross, 2007). Adaptive emotional regulation involves selecting appropriate strategies and flexibility in their application, which is an indicator of psychological adjustment. Ineffective regulation leads to maladaptive emotional, cognitive and behavioral consequences, jeopardizing the individual’s ability to adapt to the situation (Cicchetti et al., 1995; Verzeletti et al., 2016). Poorly adaptive cognitive strategies for emotional regulation have been indicated as a risk factor for depression and anxiety (Aldao et al., 2010), increasing negative thoughts and compromising problem-solving. On the other hand, adaptive strategies conceive positive interpretations and perspectives, reducing the suffering generated by a negative event (Gross, 2007).

Some authors address emotional dysregulation as a construct with multiple dimensions encompassing deficits in several areas. These areas include awareness, understanding and acceptance of emotions, the ability to implement behaviors aimed at achieving goals and inhibiting impulsive behaviors when experiencing negative emotions, flexibility in the use of strategies aimed at modelling the intensity and/or duration of emotional responses to the detriment of their suppression, and acceptance of experiencing negative emotions that allow them to achieve personal goals (Gratz et al., 2006; Gratz and Gunderson, 2006). Gratz and Roemer (2004) claim that the difficulties in emotional regulation can be subdivided into a few dimensions, such as the nonacceptance of the emotional response, the lack of awareness and misunderstanding of the emotions, the difficulties in maintaining a behavior directed toward the goals, the difficulties in controlling impulses, limited access to emotional regulation strategies and, finally, lack of emotional clarity.

Research has highlighted that, based on a wide range of psychopathological conditions, there are deficits in emotional regulation (Gratz and Roemer, 2004; Chervonsky and Hunt, 2019), namely, in depressive and anxiety disorders (e.g., Masters et al., 2018). According to Turk et al. (2005), subjects with mood and anxiety disorders have a set of difficulties in managing their own emotions, which include a limited understanding of them, difficulties in identifying negative emotions and very negative reactions toward their emotional experience. Both longitudinal and cross-sectional studies have consistently demonstrated the association between deficits in the capacity for emotional regulation and depression and anxiety in adolescents (e.g., Silk et al., 2003; Masters et al., 2018). Subjects with generalized anxiety disorder showed less emotional understanding and acceptance, less ability to regulate negative emotional experiences and higher levels of negative emotional reaction. In contrast, subjects with social phobia registered less expressiveness of positive emotions and greater difficulties describing them (Mennin et al., 2002).

The family has a prominent role, especially parents, as the main agents of socialization in developing emotional regulation capacity. In the family context, children learn to express emotions to understand the messages they transmit and their various regulatory processes (Larson et al., 2002). Additionally, the quality of family relationships is one of the factors that influence the mental health of individuals (e.g., Benetti, 2006; Herrenkohl et al., 2012; Luijten et al., 2021), so it is important to understand its role in the development and maintenance of internalizing disorders in adolescence. The environment perceived and interpreted by the various elements that make up the family—defined as the family environment—has a very significant influence on the emotional, physical, social and intellectual development and the behavior of its younger members. A positive family environment, based on effective cohesion between parents and children, support, trust, intimacy and empathetic and open communication, promotes psychological and behavioral adjustment in adolescents (López et al., 2008; Teodoro et al., 2009). Several studies have revealed that a family environment characterized by high levels of conflict and low levels of cohesion interferes negatively with the psychological adjustment and well-being of adolescents (Chang et al., 2001; Belardinelli et al., 2008; Sullivan and Miklowitz, 2010). Thus, there is a positive association between family conflict and psychopathology (Benetti, 2006; Morawska and Thompson, 2009; Teodoro et al., 2010; Herrenkohl et al., 2012), as well as between lower levels of mental disorders and higher levels of support and family cohesion (Paixão et al., 2018). Thus, previous investigations highlight the importance of positive characteristics in the family environment that can mitigate the appearance of mental disorder symptoms in adolescents, namely, a family environment with the presence of support and low levels of conflict and violence. For example, adolescents with a better quality of life also present significantly better communication with both parents, greater involvement in family activities, greater perception of support from parents, as well as a better family relationship (Guedes et al., 2022).

With specific regard to internalizing disorders, several studies have confirmed significant relationships with low levels of support (emotional and functional support received) and cohesion (emotional bond) between the various members of the family and with high levels of power differentiation, in which the older members of the family have much influence on decisions (hierarchy) and family conflict (Teodoro et al., 2014). For example, LaMontagne et al. (2022) recently found that adolescents with higher family conflict had more emotion regulation difficulties and more depressive symptoms. Of the various dimensions studied, family conflict has been the most strongly associated with internalizing problems (e.g., Francisco et al., 2016; Leusin et al., 2018).

### The Current Study

Research on well-being and mental health in adolescence still needs to be investigated, especially compared to that on mental illness. Research should focus on internalizing problems, which, day by day, acquire space in society since they are not as visible as externalizing problems. To fill some of these gaps and contribute to the definition of key areas for intervention on promoting mental health and well-being in adolescents, the present study aims to understand the role of well-being in the relationship between emotional regulation difficulties, family environment, and internalizing problems among adolescents. The specific objectives are (a) to test the mediating effect of well-being on the relationship between emotional regulation difficulties and internalizing problems; (b) to test the mediating effect of well-being on the relationship between the family environment (conflict, cohesion and support) and internalizing problems; and (c) to investigate the differences between adolescents with a higher and lower risk of presenting internalizing problems (i.e., clinical and nonclinical groups, respectively) regarding emotional regulation difficulties, family environment and well-being.

Based on the proposed goals and theoretical framework presented earlier, the following research hypotheses have been established:

*H1*. The relationship between emotional regulation difficulties and internalizing problems is mediated by the adolescents’ well-being.
*H2*. The relationship between the family environment and internalizing problems in adolescents is mediated by well-being.
*H2.1*. The relationship between conflict and internalizing problems is mediated by well-being;
*H2.2*. The relationship between cohesion and support and internalizing problems is mediated by well-being.
*H3*. There were significant differences between participants who presented a higher and lower risk of internalizing problems (i.e., clinical and nonclinical groups, respectively) in the studied variables.
*H3.1*. Adolescents in the clinical group present higher levels of emotion regulation difficulties (and each specific dimension);
*H3.2*. Adolescents in the clinical group present lower levels of well-being (and each specific dimension);
*H3.3*. Adolescents in the clinical group present higher levels of family conflict;
*H3.4*. Adolescents in the clinical group present lower levels of cohesion and support in the family.

## Materials and Methods

### Participants

A group of 723 adolescents (59.3% female) aged between 12 and 18 years (*M* = 14.70, *SD* = 1.735) participated in this study. Most of the participants (67.8%) attended the 3rd cycle of basic education (7–9th grade), and the rest attended secondary education (10–12th grade) in schools in the Greater Lisbon region (55.5%) and São Miguel Island – Azores archipelago (44.5%). Most participants (68.6%) reported never having psychological counselling, 21.2% had it in the past, and 8.6% had it currently.

Most participants came from an intact nuclear family (70.7%), 16.6% from a single-parent family, 6.1% from a stepfamily, and 5.8% from other family situations. The majority of the participants come from families of average socioeconomic level, taking into account the academic qualifications of their parents: Most of the participants’ mothers have higher education (42.1%), followed by less than compulsory schooling (32.3%) and middle schooling (25.6%); in turn, most of the fathers have less than compulsory schooling (42.5%), followed by higher education (32.1%) and middle schooling (25.4%).

### Procedure

A cross-sectional design and a convenience sample were used. The Directorate-General for Innovation and Curricular Development of the Ministry of Education and the National Data Protection Commission approved the research project. After these approvals, nine public schools were approached through individual contacts (“snowball” method) and a formal authorization request to the Board of Directors of each educational establishment. Data collection was carried out in a classroom context after obtaining explicit permission from the parents and students’ informed consent (70% adherence rate). The students completed the protocols anonymously, with the presence of the subject teacher and one of the researchers, who clarified any doubts that occurred at the time. The total response time to the questionnaires was approximately 25–30 min, with younger students requiring more time than older students.

### Measures

A Questionnaire on Personal and Sociodemographic Data was built within the scope of this study, aiming to collect information at the participant’s personal and sociodemographic level (e.g., sex, age, school year, area of residence).

The Portuguese version of the Difficulties in Emotional Regulation Scale (DERS; Gratz and Roemer, 2004; Coutinho et al., 2009) was used to assess the six domains that reflect difficulties in emotional regulation: nonacceptance of negative emotions; inability to engage in goal-driven behavior when experiencing negative emotions; difficulties in controlling impulsive behavior when experiencing negative emotions; limited access to emotional regulation strategies that are perceived to be effective; lack of emotional awareness; and lack of emotional clarity. It is a self-report scale consisting of 36 items, answered on a Likert-type scale with five points from 1 (1 being “almost never applies to me” to 5 being “applies almost always to me”). The scale has good internal consistency in its original version (*α=*0.93 on the global scale and between 0.80 and 0.89 on the subscales; Gratz and Roemer, 2004), and in the Portuguese version (*α* =0.92 on the global scale and above 0.75 in all subscales; Coutinho et al., 2009). In this study sample, Cronbach’s alpha for the total scale is 0.93 and Omega index is 0.94, and between 0.74 and 0.90 in the subscales.

The Portuguese version of the Mental Health Continuum - Short Form (MHC-SF; Keyes, 2009; Matos et al., 2010) assessed adolescents’ perceived degree of well-being. It is a self-report instrument consisting of 14 items, answered on a 5-point Likert scale (from 0 being “never” to 5 being “every day”), whose sum corresponds to the level of global well-being, and which are divided into three dimensions: emotional well-being (3 items), social well-being (5 items) and psychological well-being (6 items). The Portuguese version in use has good levels of internal consistency (*α* = 0.90 on the global scale and between 0.80 and 0.85 on the subscales; Matos et al., 2010), very similar to the original version of Keyes (2009). In the study sample, the levels of internal consistency were equally high: total well-being *α* = 0.90 and ω = 0.90; between 0.80 and 0.85 for the subscales.

The Family Climate Inventory (Teodoro et al., 2009) is a self-report instrument consisting of 22 items that assess four dimensions of the family environment on a Likert scale (from 1 “completely disagree” to 5 “completely agree”): conflict (6 items related to the aggressive, critical and conflictual relationship between family members); hierarchy (6 items that analyze power differences within the family); support (5 items that measure the emotional and material support received by members of their family), and cohesion (5 items that define the bond between family members). All subscales have adequate levels of consistency in the original version (*α* =0.72 hierarchy, *α* =0.84 conflict, *α* =0.71 support and *α* =0.82 cohesion). The Portuguese version (Francisco, 2015) consists of only three dimensions (cohesion and support; hierarchy; conflict), as the results of the factor analysis do not match the original structure, with the cohesion and support items being grouped. In the present study, the hierarchy dimension was not used. In the study sample, both subscales (conflict, cohesion and support) have good levels of internal consistency (*α* = 0.88; *ω* = 0.89).

The Portuguese version of the self-report for adolescents aged 11–17 years old of the Strengths and Difficulties Questionnaire (SDQ; Goodman et al., 1998; Fleitlich et al., 2005) was used to assess internalizing symptoms. The SDQ consists of 25 items divided into 5 scales: emotional symptoms, conduct problems, hyperactivity, peer problems, and prosocial behavior. Each scale consists of 5 items, with three answer options (0 “not true”; 1 “somewhat true”; 2 “certainly true”). Goodman et al. (2010), in an in-depth psychometric study of the SDQ, proposed an alternative factor structure, with underlying theoretical justification, combining the subscale of emotional symptoms with the peer problems subscale in a single subscale, called internalizing disorders. This approach was adopted in the present study, as other researchers have also successfully used this structure (e.g., Dickey and Blumberg, 2004). According to Goodman et al. (1998, 2010), in studies with low-risk samples, “clinical cases” can be identified by a high score on one of the four difficulty scales. Since the sample of the present study is of low risk (community sample collected in a school context), individuals who showed a score considered high in one of the two scales of difficulties—above 7 in the Emotional Symptoms and above 6 in the Peer Problems’ scales (Goodman et al., 1998)—are part of the “clinical group” with internalizing problems. Regarding the instrument’s reliability, in the original version, the internal consistency coefficients of the Emotional Symptoms subscale was *α* =0.75, and *α* =0.63 (*ω* =0.63) in this study’s sample; for the Peer Problems subscale, they were *α* =0.44 and *α* =0.57 (*ω* =0.58), respectively. The new subscale “Internalizing Problems” presents higher alpha (0.69) and omega (0.68) coefficients than each subscale separately, reinforcing the appropriateness of using this strategy.

### Data Analyses

The treatment and statistical analysis were performed using the Statistical Package for Social Sciences (SPSS version 28) and R Studio. The relationship between continuous variables included in the mediation analyses was previously explored with the Pearson correlation coefficient. In simple mediation analyses, 1,000 bootstrap samples were used with a 95% confidence interval. In all mediation analyses, the overall well-being of adolescents was considered the mediating variable, and internalizing problems was the dependent variable. The Student’s *t*-test was used to test the significant differences in the clinical and nonclinical groups. Differences were considered statistically significant when the value of *p* was less than 0.05.

## Results

**Figure 1:**
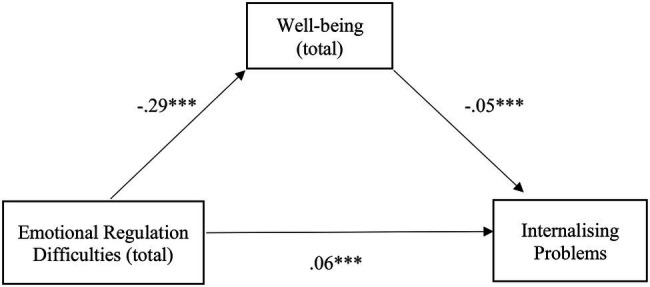
Illustration of the direct effects of emotional regulation difficulties for internalizing problems through the mediator well-being. The represented coefficients are standardized. Total effect (*β* = 0.07, *p* < 0.001). ^***^p < 0.001.

Table 1 presents the correlations between the studied variables. Regarding the mediating role of well-being, the relationship between emotional regulation difficulties and internalizing problems was partially mediated by well-being, with the indirect effect being small, although significant [*β* = 0.01, SE = 0.00, *p* < 0.001, 95% CI (0.01, 0.02)], confirming H1. The mediation model was adequate and explained 31% of the variability in internalizing problems [*F* (2, 720) = 162.53, *p* < 0.001]. Particularly, it appears that there is a negative relationship between emotional regulation difficulties and well-being, which, in turn, has a negative relationship with internalizing problems. This leads to the conclusion that the more emotional regulation difficulties are felt, the less well-being and the more internalizing problems there will be. It is also worth mentioning the positive relationship between the emotional regulation difficulties and internalizing problems (Figure 1).

**Table 1:** Correlations between all variables in study, in all participants (*N* = 723).

	1.	2.	3.	4.	5.	6.	7.	8.	9.	10.	11.	12.	13.	14.	15.
1.DERS (total)	–														
2. Strategies	**0.895** ^***^	–													
3. Non-acceptance	**0.755** ^***^	**0.671** ^***^	–												
4. Awareness	**0.366** ^***^	**0.178** ^***^	0.012	–											
5. Impulse	**0.776** ^***^	**0.630** ^***^	**0.525** ^***^	**0.207** ^***^	–										
6. Goals	**0.710** ^***^	**0.623** ^***^	**0.474** ^***^	0.006	**0.483** ^***^	–									
7. Clarity	**0.673** ^***^	**0.515** ^***^	**0.399** ^***^	**0.362** ^***^	**0.390** ^***^	**0.328** ^***^	–								
8. Well-being (total)	**−0.506** ^***^	**−0.480** ^***^	**−0.267** ^***^	**−0.409** ^***^	**−0.266** ^***^	**−0.371** ^***^	**−0.403** ^***^	–							
9. Emotional well-being	**−0.519** ^***^	**−0.516** ^***^	**−0.296** ^***^	**−0.334** ^***^	**−0.295** ^***^	**−0.321** ^***^	**−0.422** ^***^	**0.807** ^***^	–						
10. Social well-being	**−0.385** ^***^	**−0.361** ^***^	**−0.178** ^***^	**−0.291** ^***^	**−0.175** ^***^	**−0.343** ^***^	**−0.304** ^***^	**0.883** ^***^	**0.611** ^***^	–					
11. Psychological well-being	**−0.462** ^***^	**−0.408** ^***^	**−0.244** ^***^	**−0.423** ^***^	**−0.249** ^***^	**−0.286** ^***^	**−0.358** ^***^	**0.911** ^***^	**0.655** ^***^	**0.663** ^***^	–				
12. Family Conflict	**0.333** ^***^	**0.322** ^***^	**0.239** ^***^	**0.112** ^**^	**0.290** ^***^	**0.185** ^***^	**0.279** ^***^	**−0.212** ^***^	**−0.246** ^***^	**−0.137** ^***^	**−0.185** ^***^	–			
13. Cohesion and Support	**−0.262** ^***^	**−0.236** ^***^	−0.063	**−0.271** ^***^	**−0.200** ^***^	**−0.130** ^**^	**−0.221** ^***^	**0.361** ^***^	**0.331** ^***^	**0.286** ^***^	**0.338** ^***^	**−0.537** ^***^	–		
14. Internalizing Problems	**0.536** ^***^	**0.562** ^***^	**0.423** ^***^	**0.141** ^***^	**0.398** ^***^	**0.331** ^***^	**0.376** ^***^	**−0.418** ^***^	**−0.450** ^***^	**−0.313** ^***^	**−0.357** ^***^	**0.366** ^***^	**−0.261** ^***^	–	
15. Sex	**−0.192** ^***^	**−0.169** ^***^	**−0.079** ^*^	−0.046	**−0.086** ^*^	**−0.209** ^***^	**−0.223** ^***^	0.031	**0.181** ^***^	**0.187** ^***^	**0.094** ^*^	−0.010	0.042	**−0.174** ^***^	–
16. Age	−0.037	−0.034	−0.021	0.003	−0.032	0.013	0.023	−0.032	**−0.139** ^***^	**−0.103** ^**^	−0.010	**0.097** ^*^	**−0.113** ^**^	**0.081** ^*^	−0.055

^***^ p < 0.001; ^**^ p < 0.01; ^*^ p < 0.05; DERS, Difficulties in Emotion Regulation Scale.

**Figure 2:**
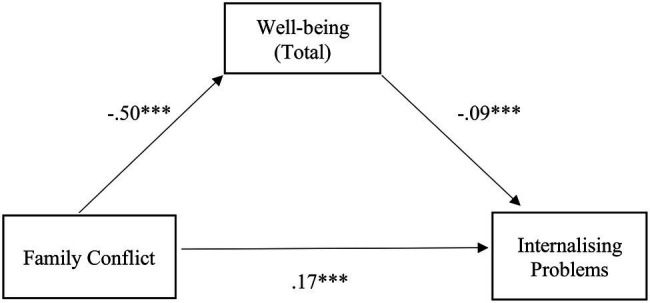
Illustration of the direct effects of the family conflict for internalizing problems through the mediator well-being. The represented coefficients are standardized. Total effect (*β* = 0.21, *p* < 0.001). ^***^p < 0.001.

The relationship between family conflict and internalizing problems was also partially mediated by well-being, with a significant indirect effect, despite being small [*β* = 0.04, SE = 0.01, *p* < 0.001, 95% CI (0.03, 0.07)], confirming H2.1. The mediation model was adequate and explained 26% of the variability in internalizing problems [*F* (2,720) = 123.42, *p* < 0.001]. Specifically, there is a negative relationship between conflict and well-being, which, in turn, has a negative relationship with internalizing problems, concluding that the greater the family conflict, the lesser the well-being, and the greater the internalizing problems. The positive relationship between conflict and internalizing problems should also be noted (Figure 2).

**Figure 3:**
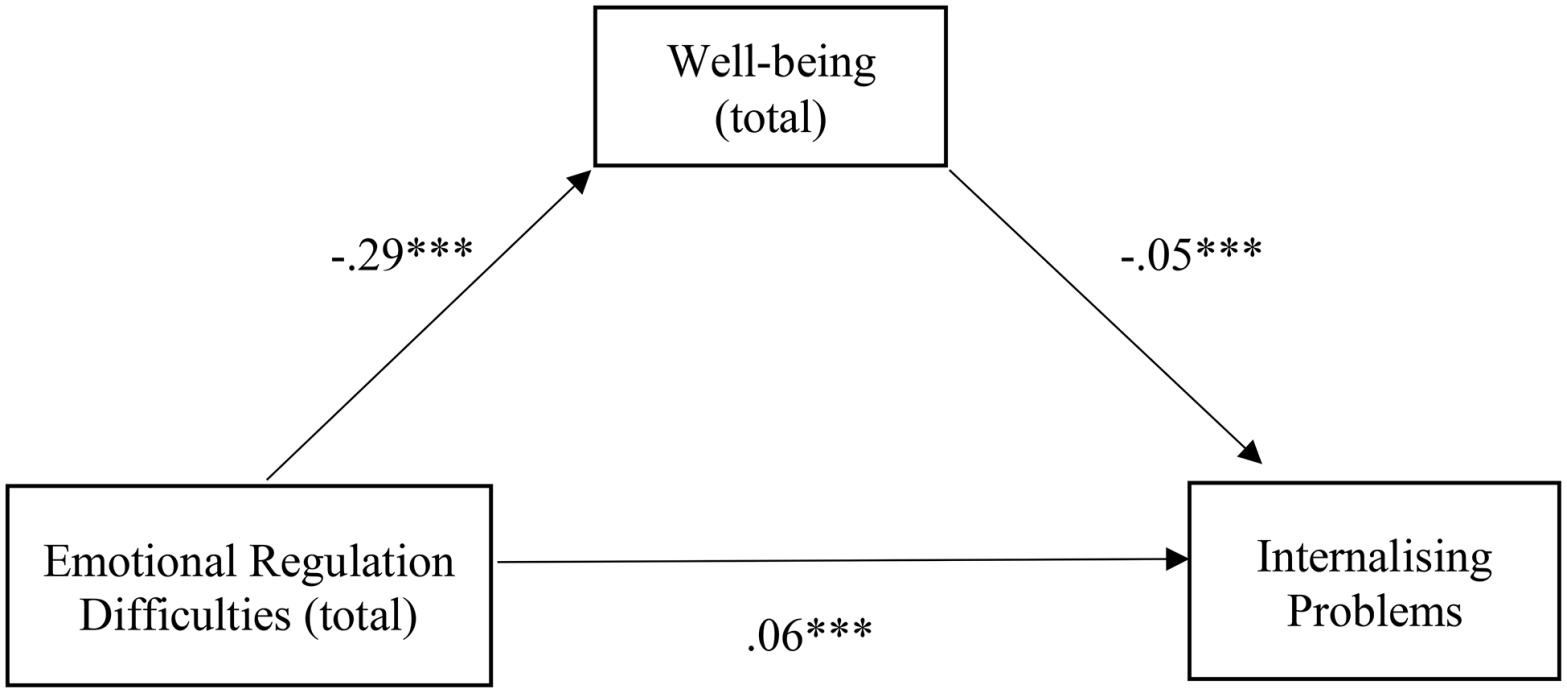
Illustration of the direct effects of family cohesion and support for internalizing problems through the mediator well-being. The represented coefficients are standardized. Total effect (*β* = −0.11, *p* < 0.001). ^***^p < 0.001.

The relationship between family cohesion and support and internalizing problems was also partially mediated by well-being, with a small, albeit significant, indirect effect [*β* = −0.06, SE = 0.01, *p* < 0.001, 95% CI (−0.08, −0.04)], confirming H2.2. The mediation model was adequate and explained 19% of the variability in internalizing problems [*F* (2,720) = 83.99, *p* < 0.001]. There is a positive relationship between family cohesion and support and well-being, which, in turn, has a negative relationship with internalizing problems, concluding that the greater the family cohesion and support, the greater the well-being, and the less the internalizing problems. Additionally, of note is the negative relationship between cohesion and support and internalizing problems (Figure 3).

Additionally, the mediation model was also tested with the three predictor variables. The model also proved to be adequate, explaining 31% of the variability in internalizing problems [*F* (4, 718) = 81.99, *p* < 0.001]. Specifically, the relationship between emotional regulation difficulties and internalizing problems was mediated by well-being. An indirect effect was small but significant [*β* = 0.01, SE = 0.00, *p* = <0.001, CI 95% (0.00, 0.01)]; the relationship between family conflict and internalizing problems was mediated by well-being, and the indirect effect was also small but significant [*β* = −0.01, SE = 0.01, *p* = <0.001, 95% CI (−0.01, 0.00)]. Nonetheless, the relationship between familiar cohesion and support and internalizing problems was not mediated by well-being, and the indirect effect was not significant [*β* = − 0.01, SE = 0.01, *p* = >0.05, 95% CI (−0.02, 0.00)].

The correlations separated for the clinical and nonclinical groups (Table 2) showed different correlations between the studied variables among the clinical group. Significant correlations (although weaker than in the nonclinical group) were found between internalizing problems and well-being, as well as between internalizing problems and family conflict. However, the correlations of internalizing problems with difficulties in emotion regulation or cohesion and support were nonsignificant. For this reason, the previous models of mediation were tested specifically for the clinical group.

**Table 2:** Correlations between all variables in study, in participants included in clinical (*n* = 130) and nonclinical groups (*n* = 584).

	1.	2.	3.	4.	5.	6.	7.	8.	9.	10.	11.	12.	13.	14.	15.	16.
1.DERS (total)	–	**0.864** ^***^	**0.708** ^***^	**0.342** ^***^	**0.747** ^***^	**0.688** ^***^	**0.632** ^***^	**−0.455** ^***^	**−0.452** ^***^	**−0.337** ^***^	**−0.422** ^***^	**0.259** ^***^	**−0.232** ^***^	**0.425** ^***^	**−0.145** ^***^	−0.013
2. Strategies	**0.896** ^***^	–	**0.610** ^***^	**0.109** ^*^	**0.590** ^***^	**0.581** ^***^	**0.431** ^***^	**−0.377** ^***^	**−0.413** ^***^	**−0.269** ^***^	**−0.324** ^***^	**0.217** ^***^	**−0.180** ^***^	**0.435** ^***^	**−0.119** ^**^	−0.041
3. Non-acceptance	**0.775** ^***^	**0.666** ^***^	–	−0.050	**0.431** ^***^	**0.441** ^***^	**0.339** ^***^	**−0.176** ^***^	**−0.200** ^***^	**−0.102** ^*^	**−0.168** ^***^	**0.162** ^***^	−0.036	**0.288** ^***^	−0.033	−0.014
4. Awareness	**0.433** ^***^	**0.278** ^**^	0.085	–	**−0.188** ^***^	−0.031	**0.346** ^***^	**−0.411** ^***^	**−0.343** ^***^	**−0.282** ^***^	**−0.422** ^***^	0.058	**−0.238** ^***^	**0.088** ^*^	−0.034	0.032
5. Impulse	**0.752** ^***^	**0.541** ^***^	**0.581** ^***^	**0.209** ^*^	–	**0.436** ^***^	**0.329** ^***^	**−0.201** ^***^	**−0.225** ^***^	**−0.125** ^**^	**−0.186** ^***^	**0.209** ^***^	**−0.165** ^***^	**0.261** ^***^	−0.034	−0.024
6. Goals	**0.666** ^***^	**0.583** ^***^	**0.363** ^***^	0.024	**0.434** ^***^	–	**0.270** ^***^	**−0.312** ^***^	**−0.246** ^***^	**−0.299** ^***^	**−0.234** ^***^	**0.127** ^**^	−0.083	**0.253** ^***^	**−0.168** ^***^	0.002
7. Clarity	**0.645** ^***^	**0.510** ^***^	**0.361** ^***^	**0.355** ^***^	**0.349** ^***^	**0.300** ^**^	–	**−0.347** ^***^	**−0.396** ^***^	**−0.256** ^***^	**−0.294** ^***^	**0.218** ^***^	**−0.194** ^***^	**0.279** ^***^	**−0.229** ^***^	0.052
8. Well-being (total)	**−0.427** ^***^	**−0.480** ^***^	**−0.251** ^**^	**−0.347** ^***^	−0.169	**−0.340** ^***^	**−0.356** ^***^	–	**. 776** ^***^	**0.868** ^***^	**0.905** ^***^	**−0.157** ^***^	**−0.328** ^***^	**−0.326** ^***^	0.016	−0.034
9. Emotional well-being	**−0.414** ^***^	**−0.471** ^***^	**−0.251** ^**^	**−0.290** ^**^	**−0.186** ^*^	**−0.284** ^**^	**−0.286** ^**^	**0.826** ^***^	–	**0.554** ^***^	**0.636** ^***^	**−0.175** ^***^	**0.290** ^***^	**−0.338** ^***^	**0.115** ^**^	**−0.116** ^**^
10. Social well-being	**−0.301** ^**^	**−0.380** ^***^	−0.160	**−0.241** ^**^	−0.054	**−0.330** ^***^	**−0.236** ^**^	**0.905** ^***^	**0.681** ^***^	–	**0.629** ^***^	**−0.110** ^**^	**0.267** ^***^	**−0.226** ^***^	**0.128** ^**^	**−0.090** ^*^
11. Psychological well-being	**−0.396** ^***^	**−0.393** ^***^	**−0.237** ^**^	**−0.358** ^***^	**−0.208** ^*^	**−0.244** ^**^	**−0.374** ^***^	**0.907** ^***^	**0.631** ^***^	**0.686** ^***^	–	**−0.136** ^**^	**0.293** ^***^	**−0.283** ^***^	0.049	0.008
12. Family Conflict	**0.250** ^**^	**0.267** ^**^	0.177	0.151	**0.240** ^**^	0.099	0.172	−0.098	−0.141	0.034	−0.101	–	**−0.480** ^***^	**0.231** ^***^	0.015	**0.106** ^*^
13. Cohesion and Support	**−0.202** ^*^	**−0.221** ^*^	−0.032	**−0.326** ^***^	**−0.160**	−0.158	−0.166	**0.341** ^***^	**0.321** ^***^	**0.222** ^*^	**0.369** ^***^	**−0.634** ^***^	–	**−0.250** ^***^	0.042	**−0.133** ^**^
14. internalizing Problems	0.158	**0.192** ^*^	**0.253** ^**^	0.101	**0.233** ^**^	−0.064	0.097	**−0.201** ^*^	**−0.211** ^*^	−0.140	−0.164	**0.224** ^*^	−0.015	–	**−0.148** ^***^	**0.090** ^*^
15. Sex	−0.121	−0.089	−0.022	−0.006	−0.048	**−0.246** ^**^	−0.007	−0.033	**0.232** ^**^	**0.275** ^**^	0.104	0.159	−0.103	**0.193** ^*^	–	−0.048
16. Age	−0.138	−0.070	−0.071	−0.143	−0.100	0.088	−0.146	−0.099	**−0.237** ^**^	−0.133	−0.035	0.071	−0.026	0.093	−0.075	–

Values for participants included in clinical group are in the lower-left triangle and values for participants included in nonclinical group are in the upper-right triangle. ^***^p < 0.001; ^**^p < 0.01; ^*^p < 0.05; DERS, Difficulties in Emotion Regulation Scale.

**Figure 4:**
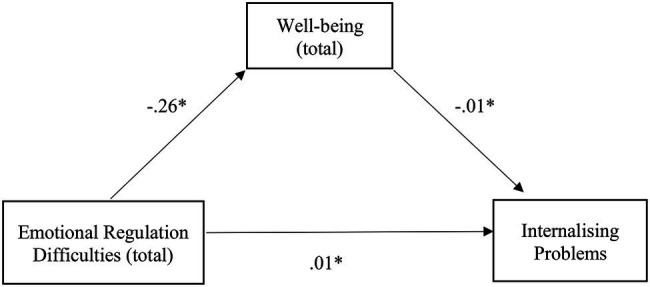
Illustration of the direct effects of emotional regulation difficulties for internalizing problems through the mediator well-being in clinical group. The represented coefficients are standardized. Total effect (*β* = 0.01, *p* < 0.05). ^*^p < 0.05.

The results revealed that the relationship between emotional regulation difficulties and internalizing problems was mediated by well-being, with a small yet significant, indirect effect [*β* = 0, SE = 0.01, *p* = <0.05, 95% CI (0.00, 0.01)]. The mediation model was inadequate, explaining only 5% of the variability in internalizing problems [*F* (2, 127) = 3.59, *p* < 0.05]. Nonetheless, a negative relationship exists between emotional regulation difficulties and well-being, negatively related to internalizing problems. This leads to the conclusion that the more emotional regulation difficulties are felt, the less well-being and the more internalizing problems there will be. It is also worth mentioning the positive relationship between emotional regulation difficulties and internalizing problems (Figure 4).

**Figure 5:**
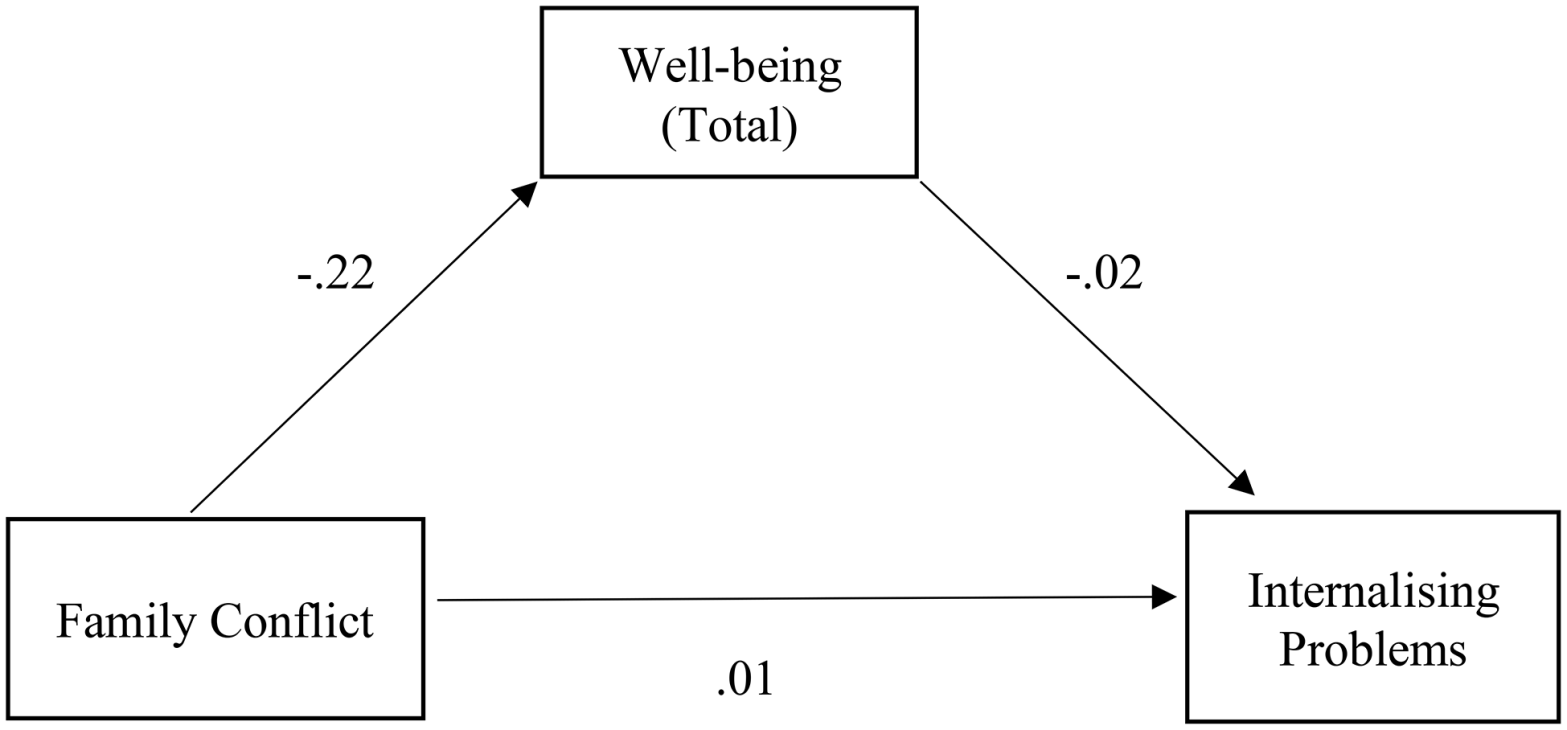
Illustration of the direct effects of the family conflict for internalizing problems through the mediator well-being in clinical group. The represented coefficients are standardized. Total effect (*β* = 0.01, *p* > 0.05).

**Figure 6:**
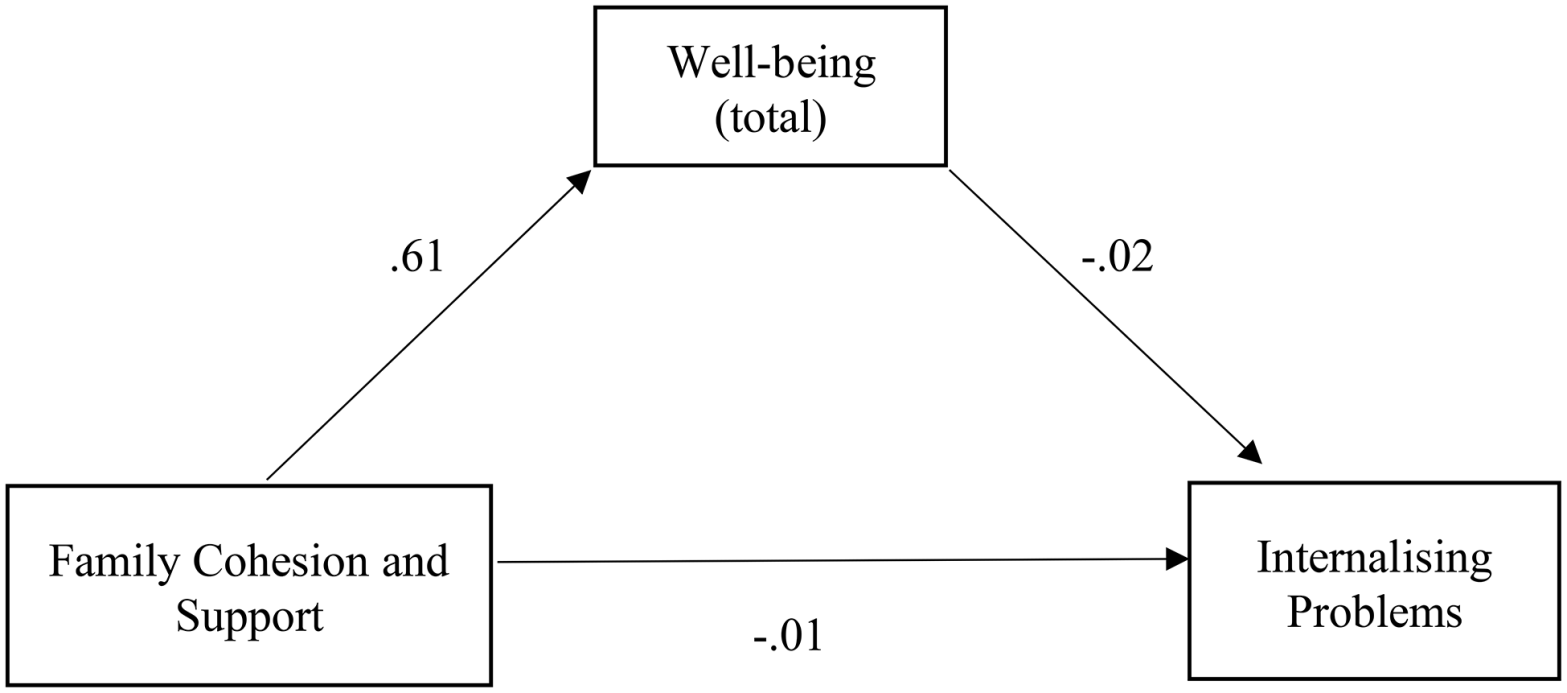
Illustration of the direct effects of family cohesion and support for internalizing problems through the mediator well-being in clinical group. The represented coefficients are standardized. Total effect (*β* = 0.02, *p* > 0.05).

Considering the family variables, the mediation model revealed that the relation between family conflict and internalizing problems was not successfully mediated by well-being (Figure 5), with the indirect effect not being significant [*β* = 0, SE = 0.02, *p* > 0.05, 95% CI (0.00, 0.01)]. The relationship between cohesion and support and internalizing problems was also not mediated by well-being (Figure 6) with a nonsignificant indirect effect [*β* = −0.01, SE = 0.02, *p* > 0.05, 95% CI (−0.03, 0.00)].

Table 3 presents the comparison results between the clinical and nonclinical groups for the study variables. All differences were considered statistically significant, confirming hypotheses H3.1, H3.2, H3.3, and H3.4. Specifically, the clinical group revealed higher values regarding total emotional regulation difficulties and their specific dimensions. The nonclinical group showed higher values of total well-being and its respective dimensions. For the family environment, the clinical group revealed higher conflict values and lower values of cohesion and support.

**Table 3:** Comparison between clinical and nonclinical groups regarding emotional regulation difficulties, well-being, conflict, cohesion and support (*n* = 714).

	Clinical Group (*n* = 130)	Nonclinical Group (*n* = 584)		
	*M (SD)*	*M (SD)*	*df*	*t*
Strategies	25.92 (8.24)	16.54 (6.78)	165.79	**−11.96** ^***^
Non-acceptance	16.96 (6.38)	11.92 (5.29)	167.94	**−8.33** ^***^
Awareness	16.73 (5.14)	15.38 (4.73)	700	**−2.90** ^**^
Impulses	16.18 (5.99)	11.68 (4.98)	166.96	**−7.89** ^***^
Goals	18.17 (4.84)	14.46 (5.03)	693	**−7.61** ^***^
Clarity	14.15 (4.09)	10.98 (3.87)	695	**−8.23** ^***^
DERS (total)	107.22 (25.10)	80.90 (20.89)	152.24	**−10.52** ^***^
Emotional well-being	11.83 (3.69)	14.57 (2.66)	157.34	**7.95** ^***^
Social well-being	14.32 (6.33)	17.95 (5.44)	166.76	**5.95** ^***^
Psychological well-being	21.17 (6.95)	25.38 (6.04)	168.84	**6.30** ^***^
Well-being (total)	47.20 (15.01)	57.97 (12.30)	158.54	**7.40** ^***^
Conflict	15.54 (6.53)	11.25 (5.06)	158.14	**−6.90** ^***^
Cohesion and Support	37.21 (8.46)	40.35 (7.22)	166.35	**3.86** ^***^

df, degree of freedom; ^***^p < 0.001; ^**^p < 0.01; DERS, Difficulties in Emotion Regulation Scale.

## Discussion

The main objectives of this study were to understand the mediating effect of well-being on the relationship between emotional regulation difficulties and the internalizing problems, and in the relationship between the family environment (conflict, cohesion and support) and internalizing problems. Moreover, it aimed to test the differences between adolescents with a higher and lower risk of internalizing problems (e.g., clinical and nonclinical groups, respectively) concerning emotional regulation difficulties, family environment and well-being.

### The Mediating Role of Well-Being

The results of the present study revealed that well-being has a partial mediating effect on the relationship between emotional regulation difficulties and internalizing problems. Considering the overall path of the direct effects obtained, the more emotional regulation difficulties are felt, the lower the well-being and the greater the Internalizing problems. Previous studies have already found that individuals who did not have difficulties in emotional regulation and used adaptive strategies had higher levels of well-being, manifesting happiness and satisfaction with life (e.g., Freire and Tavares, 2011). Likewise, greater difficulties in emotional regulation experienced by adolescents are also identified in the literature as being associated with Internalizing problems (Silk et al., 2003; Coutinho et al., 2009; Verzeletti et al., 2016) and with suicide ideation (Swee et al., 2020). Swee et al. (2020) identified belongingness— a relevant part of the well-being concept—as a mediator in the relationship between ﻿dysfunctional emotional regulation and suicidal ideation, thus demonstrating the need to study the mediating role of well-being and its importance in the psychological adjustment of adolescents. Since research on the mediating effect of well-being in this relationship is scarce, these results provide new knowledge for treating and understanding socio-emotional maladjustment in adolescents. Although emotional regulation difficulties are related to Internalizing problems, there may be a reduction in this impact by developing skills and strategies that promote global well-being in adolescents (Brenning et al., 2021).

Well-being also had a partial mediating effect on the relationship between the family environment and Internalizing problems. Considering the direct effects, higher levels of family conflict are associated with less well-being among adolescents, which, in turn, is associated with greater Internalizing problems. Likewise, high levels of cohesion and support within the family act as enhancers of well-being, which is then reflected in fewer Internalizing problems in adolescents. Previous studies have already shown that a family environment characterised by high levels of conflict and low levels of cohesion negatively interfere with the well-being, quality of life and psychological adjustment of adolescents (Belardinelli et al., 2008; Sullivan and Miklowitz, 2010; Cunsolo, 2017; Guedes et al., 2022), namely, in the rise of Internalizing problems (Teodoro et al., 2014; Paixão et al., 2018).

Considering only the clinical group, even though it was observed that well-being partially mediated the relationship between emotional regulation difficulties and internalizing problems, it was not as robust as when compared with the results described for the global sample. It was also noted that this group presented no significant mediation between the family environment (specifically family conflict and cohesion and support) and internalizing problems. However, these results can be explained by the small number of participants integrated into the clinical group, so future studies should further explore these possible effects. Nevertheless, the present study results allow a more in-depth understanding of the relationships between these constructs among adolescents in general, as the association found between the family environment (conflict and cohesion and support) and internalizing problems can change with the influence of well-being. In this sense, the promotion of the global well-being of adolescents will influence the relationship between family conflicts and the psychosocial adjustment, reducing the development of internalizing problems.

### Risk Associated With Internalizing Problems

Adolescents at higher and lower risk of internalising problems showed significant differences in all studied variables. According to previous research, the greatest difficulties in emotional regulation reported by adolescents in the clinical group were expected. Studies have shown that individuals from the clinical population, namely, those with mood and anxiety disorders, had difficulties in managing their own emotions, as well as a limited understanding of them. They also presented difficulties in identifying negative emotions, controlling them, following goal-driven behaviors when they experience negative emotions, and presenting negative reactions toward their emotional experience (e.g., Turk et al., 2005; Coutinho et al., 2009).

Regarding the family environment, adolescents in the clinical group had higher levels of conflict and lower levels of family cohesion and support. Previous studies with adolescents with internalizing and externalizing problems reached the same conclusions (Teodoro et al., 2014), suggesting that the family environment is relevant to adolescents’ socioemotional adjustment. Given that a positive family environment assumes, as a basis, affective cohesion between parents and children, support, trust, empathic communication and openness, these can be considered protective factors of the children’s psychological adjustment. In this sense, a critical and aggressive relationship can be associated with high levels of conflict and, in turn, with adolescents’ internalizing problems (López et al., 2008).

Finally, the adolescents in the clinical group presented lower levels of well-being, which is in line with the results of previous studies that compared adolescents with and without anxiety and depression (Derdikman-Eiron et al., 2011; Luijten et al., 2021). In this regard, it is important to understand that, according to Keyes (2002), low levels of subjective well-being are associated with a less positive assessment of the overall satisfaction with one’s life (emotional well-being), with a more negative assessment of the quality of relationships with others and with the environment (social well-being), and even with lower personal acceptance and sense of life (psychological well-being). These are, in fact, aspects also frequently found in adolescent patients with internalizing problems (Khazanov and Ruscio, 2016), which reveals the relative overlap between the mental health and mental illness continuum.

### Strengths, Limitations, and Implications for Future Studies

There is no prior research on the mediating effect of well-being on the analyzed constructs and internalizing problems, so these innovative results provide new knowledge regarding socioemotional (dis)adjustment in adolescents. Most of the current well-being research focuses on mediating constructs that influence well-being (e.g., Huang et al., 2018; Roemer and Harris, 2018; Yu and Luo, 2018) and not well-being as a mediator. However, according to the existing literature, this problem still needs greater in-depth research at the level of internalizing problems in adolescents (Zalk, 2020).

New implications are also drawn for the study of emotional regulation difficulties. They play a critical role in developing and maintaining internalizing problems in adolescents, making it extremely important to identify and understand the deficits that promote emotional dysregulation (Schäfer et al., 2016). Likewise, it is important to highlight the selection of appropriate strategies and flexibility in their application for adaptive emotional regulation since maladaptive emotional regulation strategies are indicated as risk factors for developing most mental disorders (Aldao et al., 2010).

Some limitations of the present study should be mentioned. First, the sampling was neither random nor representative of the Portuguese population, not allowing the generalization of the results. Second, using self-report instruments could have introduced some skewing on the answers regarding social desirability and randomness. Third, the small sample size of the clinical group and the way it was constituted. Actually, in this study, the clinical group refers to the group of participants who had a high score on one of the Emotional Symptoms and/or Relationship Problems with Colleagues subscales (*cf*. Goodman et al., 2010) and not in the sense that some type of clinical diagnosis was made or reported. Finally, the study’s cross-sectional nature does not allow us to make definitive statements regarding the directionality or causality of the associations.

Future studies should explore the influence of other factors contributing to the subjective well-being of adolescents, as well as the role of well-being as a mediator of the relationship between other variables and internalizing problems, such as the quality of family communication or coping strategies commonly used by adolescents to deal with the challenges of this developmental stage. In addition to longitudinal studies, which will allow verification of the stability of the studied relations, it will also be important to replicate this study comparing adolescents without and with a medical diagnosis of internalizing disorders. In this case, data collection should occur in institutions used by adolescents with difficulties (e.g., primary healthcare centers, hospitals), and not in the school community. Furthermore, it would be of great interest to address the results of the present study from a qualitative perspective, for example, including interviews with adolescents about the way they use different emotion regulation strategies in various situations, to better understand its relationships with the various dimensions of well-being and internalizing problems.

## Implications for Practice

These results may be relevant to the intervention with adolescents, both in terms of preventing internalizing problems and promoting their well-being in psychotherapeutic intervention. In the context of clinical intervention, it will be relevant to promote more adaptive emotional regulation strategies to help conceptualize the difficulties and find additional viable alternatives for self-regulation. A recent meta-analysis demonstrated the effectiveness of existing interventions to improve emotion regulation, with improvements in psychopathology in youth (Moltrecht et al., 2021). At the same time, it is essential to promote belongingness to a group and create appropriate supportive relationships, including family relationships characterized by high cohesion and support and few conflicts. In addition, a recent study with Portuguese parents and adolescents suggested that mindful parenting interventions might be useful to foster adaptive emotion regulation in children by facilitating their self-compassion and psychological flexibility (Moreira and Canavarro, 2020). Interventions with both parents and adolescents can have important outcomes in reducing adolescent internalizing problems.

The results also provide relevant information for the design of psychopathology prevention programs in adolescents and the promotion of mental health and global well-being. For example, Johnstone et al. (2020) proved the efficacy of universal school-based prevention programs for anxiety and depression symptomatology in children and adolescents by promoting emotion regulation strategies. Positive psychology is also extremely relevant to assist in the understanding and development of high levels of psychological well-being in students, staff and school, considering that a positive school environment can help solve many problems (Duckworth et al., 2009; Borkar, 2016). Thus, a focus on different dimensions, such as emotional literacy, awareness of different emotions, emotional expression and differentiation, seems relevant to facilitate the adoption of adjusted attitudes and behaviors in intensely emotional situations. Furthermore, this will enhance the sense of belongingness to a group, promote interpersonal knowledge, provide problem-solving strategies, and promote positive family interactions.

## Data Availability Statement

The raw data supporting the conclusions of this article will be made available by the authors upon request, without undue reservation.

## Ethics Statement

The studies involving human participants were reviewed and approved by CRC-W: Católica Research Centre for Psychological, Family and Social Wellbeing, from Universidade Católica Portuguesa. Written informed consent to participate in this study was provided by the participants’ legal guardian/next of kin.

## Author Contributions

BR designed the study, collected, managed and analyzed data, and wrote the draft of this manuscript. RF designed the study, supervised data collection and data analyzes, reviewed the draft, and contributed to the final version of the manuscript. All authors contributed to the article and approved the submitted version.

## Conflict of Interest

The authors declare that the research was conducted in the absence of any commercial or financial relationships that could be construed as a potential conflict of interest.

## Publisher’s Note

All claims expressed in this article are solely those of the authors and do not necessarily represent those of their affiliated organizations, or those of the publisher, the editors and the reviewers. Any product that may be evaluated in this article, or claim that may be made by its manufacturer, is not guaranteed or endorsed by the publisher.
